# Experiences of the Molecular Diagnosis of Fragile X Syndrome in Ecuador

**DOI:** 10.3389/fpsyt.2021.716311

**Published:** 2021-12-13

**Authors:** Juan Pozo-Palacios, Arianne Llamos-Paneque, Christian Rivas, Emily Onofre, Andrea López-Cáceres, Jenniffer Villareal

**Affiliations:** ^1^Facultad de Ciencias Médicas, Escuela de Medicina, Universidad de Cuenca, Cuenca, Ecuador; ^2^Medical Genetic Services, Hospital de las Fuerzas Armadas, Quito, Ecuador; ^3^Sciences of Life Faculty, School of Dentistry, International University of Ecuador, Quito, Ecuador; ^4^Neurodesarrollo Quito, Quito, Ecuador; ^5^Fundación Santa Fe de Bogotá, Bogotá, Colombia

**Keywords:** dynamic mutation diseases, fragile X syndrome, FMR1, repetitions, intellectual disabilities

## Abstract

Fragile X syndrome (FXS) is the most common cause of hereditary intellectual disability and the second most common cause of intellectual disability of genetic etiology. This complex neurodevelopmental disorder is caused by an alteration in the CGG trinucleotide expansion in fragile X mental retardation gene 1 (*FMR1*) leading to gene silencing and the subsequent loss of its product: fragile X mental retardation protein 1 (FMRP). Molecular diagnosis is based on polymerase chain reaction (PCR) screening followed by Southern blotting (SB) or Triplet primer-PCR (TP-PCR) to determine the number of CGG repeats in the *FMR1* gene. We performed, for the first time, screening in 247 Ecuadorian male individuals with clinical criteria to discard FXS. Analysis was carried out by the Genetics Service of the Hospital de Especialidades No. 1 de las Fuerzas Armadas (HE-1), Ecuador. The analysis was performed using endpoint PCR for CGG fragment expansion analysis of the *FMR1* gene. Twenty-two affected males were identified as potentially carrying the full mutation in FMR1 and thus diagnosed with FXS that is 8.1% of the sample studied. The average age at diagnosis of the positive cases was 13 years of age, with most cases from the geographical area of Pichincha (63.63%). We confirmed the familial nature of the disease in four cases. The range of CGG variation in the population was 12–43 and followed a modal distribution of 27 repeats. Our results were similar to those reported in the literature; however, since it was not possible to differentiate between premutation and mutation cases, we can only establish a molecular screening approach to identify an expanded CGG repeat, which makes it necessary to generate national strategies to optimize molecular tests and establish proper protocols for the diagnosis, management, and follow-up of patients, families, and communities at risk of presenting FXS.

## Introduction

Fragile X syndrome (FXS) is the most common cause of hereditary intellectual disability (1). The syndrome has a complex clinical course, and patients show a unique phenotype that includes an elongated face, pointed ears, high palate, hyperlaxity, etc. Children with FXS also exhibit behavioral characteristics including anxiety, irritability, aggressiveness, and compulsivity (2). In approximately 60% of cases, patients are also diagnosed with autism spectrum disorder (ASD) (3), representing the most common monogenic cause of ASD. Autism, intellectual disability, and behavioral disorders develop at a lower rate and to a much lesser degree in females than in males (4).

FXS is caused by an abnormal expansion of a CGG triplet, greater than 200 repeats in the promoter region of the *FMR1* gene located at Xq27.3 (5, 6). This expansion causes a loss of function that prevents the encoding of FMRP, a protein involved in several brain development and function processes (7). The *FMR1* gene contains 17 exons (38kb) and the mutation normally occurs in intron 10, which contains the gene's unstable region (8). According to the reported expansion number, the allele can be classified into normal alleles, with 6–44 CGG repeats, premutation (PM) alleles, with 55–200 CGG repeats, and full mutation (FM) alleles with more than 200 CGG repeats. Intermediate or gray zone alleles are those harboring between 45 and 54 CGG repeats and are considered precursors of the PM allele (9).

Methods to diagnostic FXS include molecular tests such as PCR that allows determining if there is a CGG expansion, which, in combination with SB, allows determining the exact number of CGG repeats and the degree of methylation of the *FMR1* gene (10), and triplet-primed PCR assay that detects *FMR1* alleles throughout the expanded range (11). On the other hand, immunohistochemical tests can be performed to determine the presence or absence of the FMRP protein (12).

The frequency for the FM has been estimated to be higher in males than in females; internationally, the prevalence is 1/4,000 in males and 1/8,000 in females, and the PM frequency for this gene is 1/259 in females and 1/379 in male carriers (13). There is some variation depending on the population studied, e.g., in the United States, the prevalence of the FM in European descendants is 1/3,717, and in African descendants, the prevalence is 1/2,545 males (14, 15). The prevalence of carriers of the PM in females is 1/151, and for males, it is 1/468, while for carriers of intermediate or “gray zone” alleles, it is 1/35 in females and 1/42 in males (16).

The prevalence of FXS in Ecuador remains uncertain. Most diagnoses of FXS have been made in isolation through screening projects. Of Ecuador's 17 million inhabitants, 2.72% (472,213 inhabitants) are known to have some type of disability, of which 23% (108,588 inhabitants) are cognitive or intellectual (17). The elevated number of undiagnosed patients with intellectual disabilities in our country highlights the need to implement molecular study tools to characterize the population in order to improve clinical assessment and genetic counseling for patients and their families. This work describes the experience in the molecular diagnostic approach of fragile X in the HE-1 in Ecuador.

## Materials and Methods

This is a genetic, descriptive, cross-sectional, retrospective study of 247 male patients to rule out FXS, conducted at the Genetic Service of HE-1 in Quito, Ecuador. These patients were referred for genetic evaluation between 2011 and 2021, from different institutions belonging to the Comprehensive Public Health Network.

Clinical and genetic data were extracted from the patients' physical medical records, the clinical notes reported in the HE-1 hospital management system, the referral sheets, and the record books of the molecular biology laboratory. Excel spreadsheets were used for data processing, followed by a descriptive analysis using SPSS to obtain measures of central tendency for the quantitative variables and the absolute frequency, relative frequency, and relative percentage frequency for qualitative variables.

### Participant Inclusion Criteria

Men with a diagnosis of intellectual disability, ASD, language delay, or a positive family history of FXS.

### DNA Extraction

DNA used for PCR amplification was extracted from peripheral blood leukocytes by High Pure PCR Template Preparation Kit (Roche GmbH, Mannheim, Germany) according to the manufacturer's protocol. DNA quantification was performed to ensure the quality of the DNA extraction and to determine if the nucleic acid concentration was optimal for the study (~50 ng).

### PCR-Based Method

Amplification of normal and expanded alleles was performed by endpoint PCR using the Veriti Thermal Cycler (Applied Biosystems Inc., California, USA).

Fast Start High Fidelity PCR System Kit (Roche Gmb., Mannheim, Germany) was used to assemble the PCR and two sets of oligonucleotide primers were used to amplify the repetitive CGG region of the *FMR1* gene: Fx-C: GCTCAGCTCCGTTTCGGTTTCACTTCCGGT and Fx-F: AGCCCCGCACTTCCACCACCAGCTCCTCCA, owned by Gene Link Inc. (New York, USA).

The PCR assay was performed in a 48-μl reaction volume containing about 200 ng of genomic DNA, 18 mmol/L of MgCl_2_, 0.625 U/ml of DMSO, 0.36 mmol/L of dNTPs, and 0.83 μmol/L of the pair of primers for PCR amplification of the CGG repetitive region of the *FMR1* gene (Gene Link, INC, USA) and 5 units/reaction of Taq polymerase (ROCHE; Hamburg, Germany). The reactions were denaturated for 10 min at 98°C, after which the Taq Polymerase was added; followed by 10 cycles consisting of 97°C for 30 s, 65°C for 45 s, and 68°C for 4 min; and 20 cycles consisting of 97°C for 30 s, 65°C for 45 s, 68°C for 4 min 20 s, and finally a 10-min extension at 68°C (Table 1). The PCR-amplified products were separated by 3% agarose gel electrophoresis using a 50-bp DNA Step Ladder as a molecular marker, Blue/Orange Loading Dye [6X] in the loading mix, and fluorescent Diamond Nucleic Acid Dye (Promega, Wisconsin, USA).

**Table 1:** Endpoint PCR program for fragile X syndrome.

**Step**	**Time and temperature**	**Cycles**
Denaturation	10 min at 98°C	1
Hold	5 min (pause) at 98°C	1
Add Taq enzyme mix while on hold
Denaturation	30 s at 97°C	10
Annealing	45 s at 65°C	
Extension	4 min at 68°C	
Denaturation	30 s at 97°C	20
Annealing	45 s at 65°C	
Extension	4 min 20 s at 68°C	
Fill up	10 min at 68°C	1
Hold	Hold for infinity at 4°C	1

## Results

### Resource Identification Initiative

**Figure 1:**
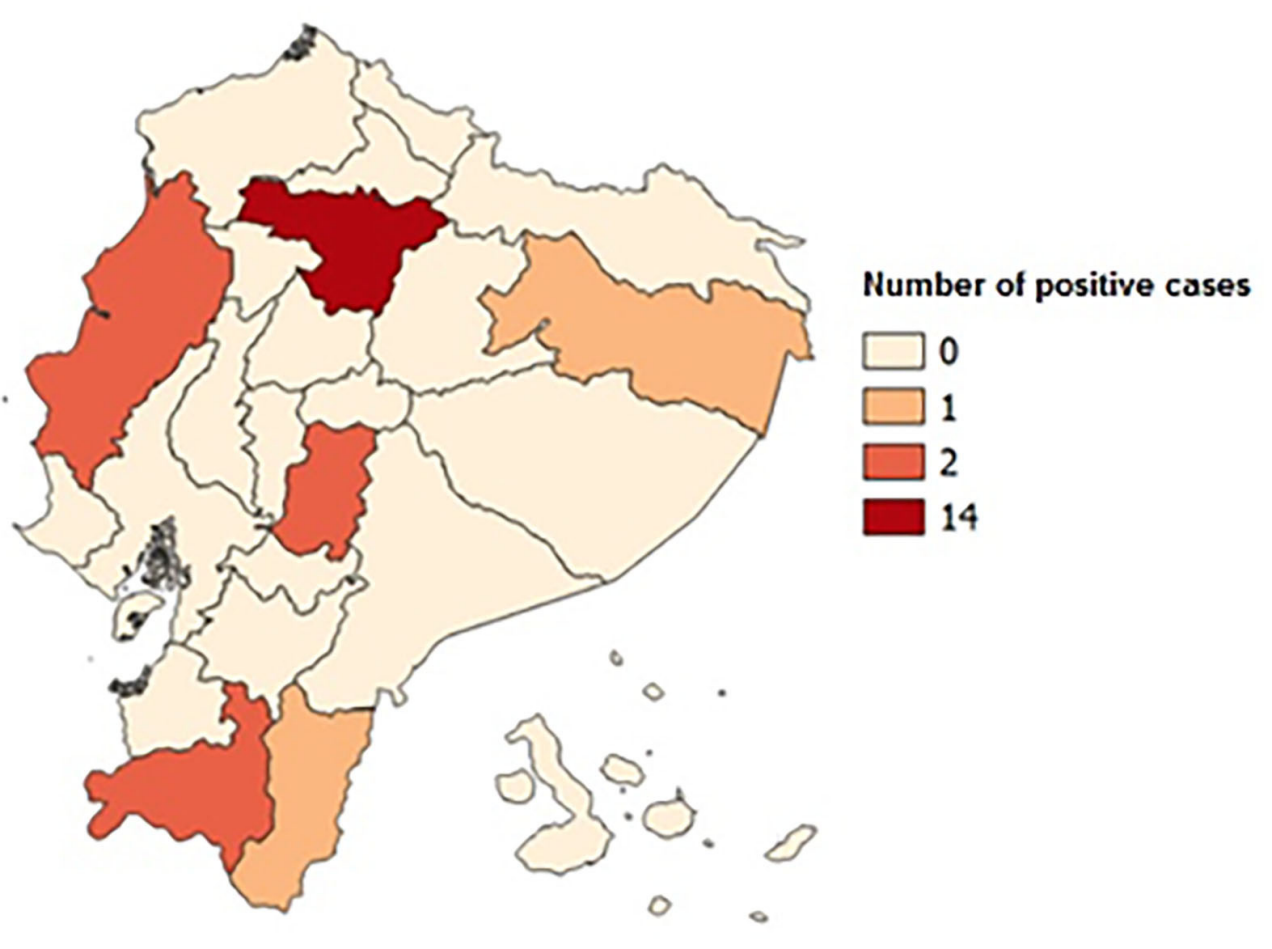
Map of Ecuador with positive case distribution by provinces. The Pichincha Province has 14 positive cases; the Guayas, Loja, and Chimborazo Provinces have 2; and the Orellana and Zamora Chinchipe Provinces have 1 positive case.

Two hundred and forty-seven men referred for genetic evaluation were screened for FXS. Of these, 22 patients were identified to have an abnormal CGG expansion of the FMR1 gene (8.1%); 46% came from the Pichincha Province (Table 1). Family history of the condition was detected in 10 patients (four families). The average age of diagnosis of the positive cases was 13 years, with age range between 2 and 31 years old. Cases were found in 6 of Ecuador's 24 provinces, with the Pichincha Province, capital of Ecuador, presenting the highest number of positive results: 14 cases, 63.63% (Figure 1). The average expansion and the number of CGG repeats for male Ecuadorian patients, with some clinical criteria studied to discard FXS, were 12–43 (**Figure 3**).

**Figure 2:**
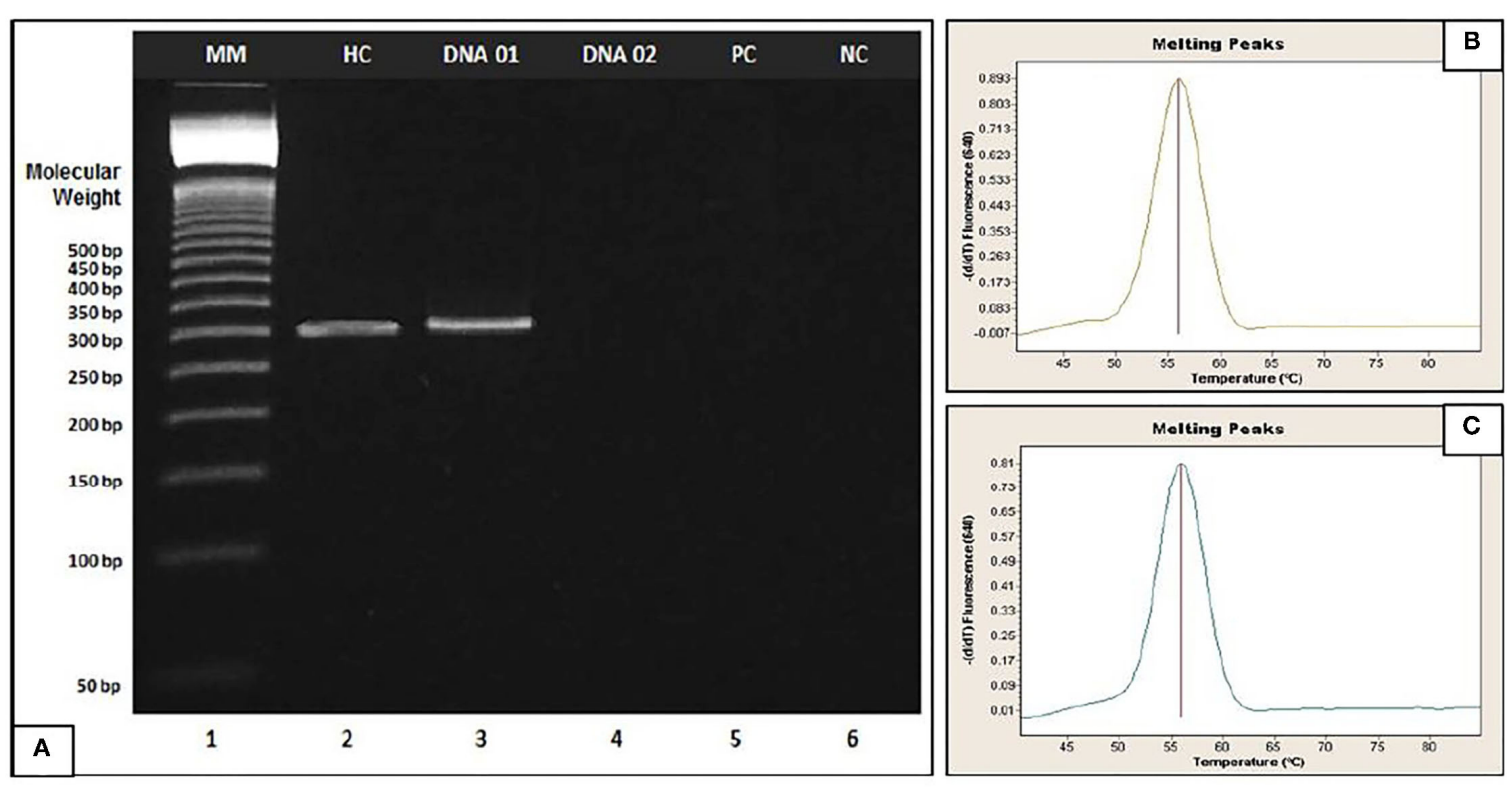
**(A)** Agarose gel electrophoresis of fragile X. Example applied to patients (DNA 01 and DNA 02). Lane 1 (MM): 50-bp DNA Step Ladder, Lane 2: Healthy Control (HC), Lane 3 (DNA 01): Healthy patient, Lane 4 (DNA 02): Affected patient, Lane 5: Positive Control (PC) and Lane 6: Negative Control (CN). **(B,C)** Amplification of genetic material by Real-Time PCR of DNA 02 and Positive Control respectively; DNA 02 and Positive control were quantified in Quantus Fluorometer (Promega, Wisconsin, USA) and concentration values between 65 and 75 ng/μl were obtained.

The absence of DNA fragments in the agarose gel indicates that the allele was so expanded that it cannot be observed given the limited sensitivity of the technique (100–120 CGG repeats). This corresponds to a positive result, which is confirmed considering the positive clinical elements (family history, intellectual disability, attention deficit and hyperactivity, autism spectrum, long narrow face, prominent jaw and ears, and joint hypermobility). In addition, this result is validated by performing real-time PCR to ensure adequate amplification of the genetic material (Figure 2).

### Translation Results

**Figure 3:**
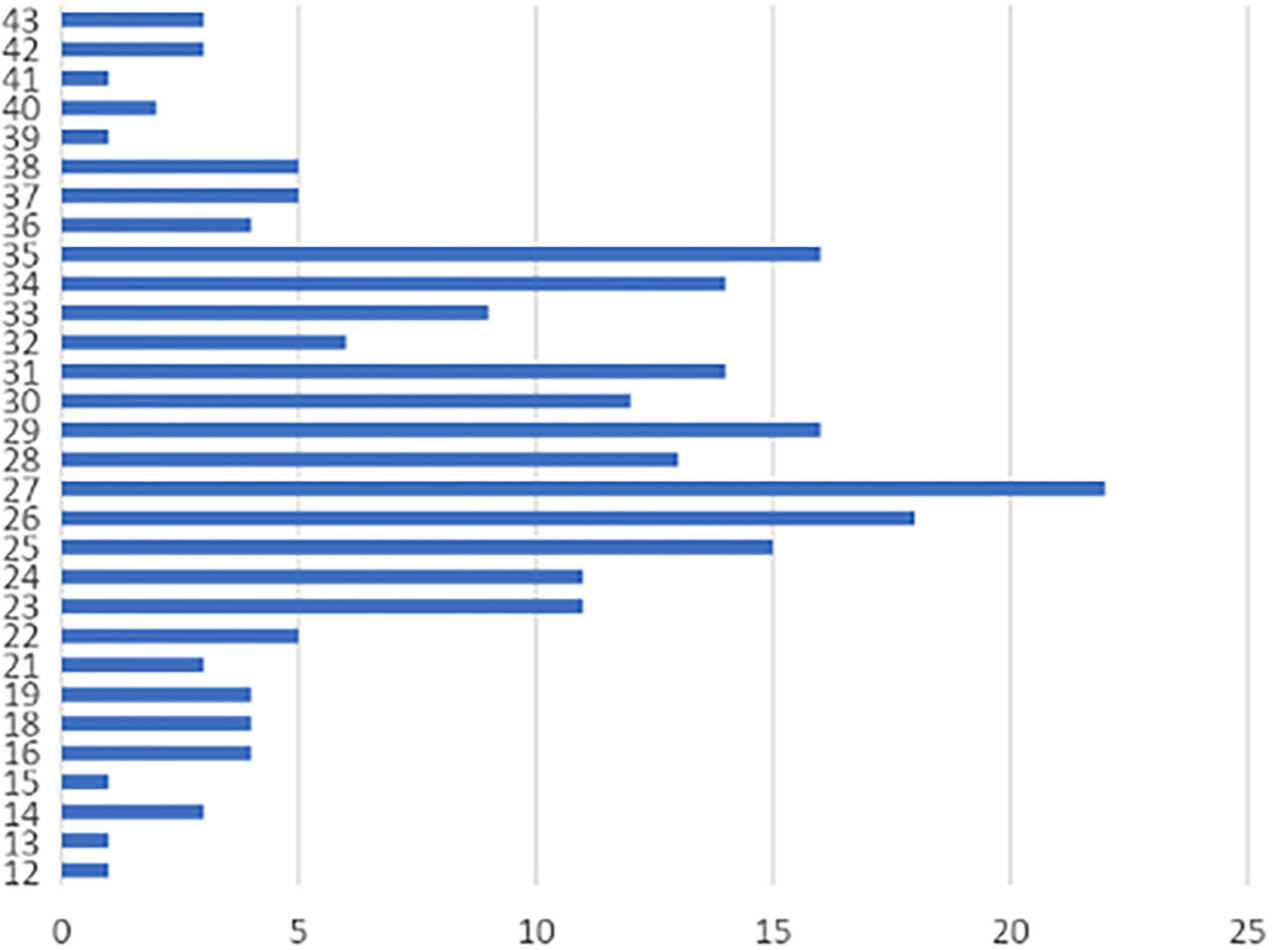
Analysis of CGG repeats in the normal unaffected population of Ecuador. Horizontal axis (X axis): CGG repeats, Vertical axis (Y axis): Frequency in the number of CGG repetitions. The range of variation of CGG in the population is 12–43 and shows a modal distribution of 27 repeats (8.90% of case). Other important percentages of frequency were of 26 repeats (7.28%), and 29 and 35 repeats (7.04%).

The average range of CGG repeats in individuals with a normal expansion in FMR1 was 27 repeats (Figure 3). PM is not included since the technique does not allow establishing the exact number of repeats in overexpanded affected alleles. The protocol only allows a normal repetition range to be established. If the value exceeds that range, the allele is not observed in the electrophoretic run, ensuring its overexpansion but without exact determination of the size.

## Discussion

FXS is the most common monogenic condition underlying ASD and intellectual disability (18). For this reason, the American College of Medical Genetics and Genomics recommends FXS testing for all individuals with developmental delay, intellectual disability, and/or behavioral problems (19).

Estimation of prevalence varies depending on the population studied and the diagnostic test used. Of the 247 Ecuadorian patients with intellectual disabilities and/or behavior problems who participated in this study, 8.1% were found to be positive through the application of molecular screening of PCR, establishing a reference point in our country, compared to previous studies that describe the presence of this condition in 7% to 8% of children with ASD (20).

Family segregation was identified in 4 families, which is justified by the inheritance mechanism, especially if a recurrent family history, an X-linked pattern, or a phenotype compatible with this disorder is considered (21). The average age of diagnosis in the patients seen in the laboratory was 13 years, much older than the age of diagnosis described in other studies, such as that described by Bailey et al., where the average age of diagnosis of FXS in children remained relatively stable over the 7-year period, at ~35 to 37 months (22). Among the studies carried out in the region, Colombia and Chile also show a higher age of diagnosis than expected (23, 24). The difference found in our data and in neighboring countries may be explained by the limited access to the health system and the lack of national protocols for molecular study of this condition in people with intellectual disabilities.

Most of the positive results were found in the Pichincha province in the Andes, possibly because it is the region of the country with the largest number of tertiary hospitals with access to molecular testing. In an ancestry analysis study in the Ecuadorian population carried out by Zambrano and collaborators, Ecuador's genetic composition is described as a mixture of three groups of ancestors: Native Americans—the largest proportion in all regions (over 50%)—and a lower proportion of people of European and African descent. The proportion of Native American ancestry in the Andes region is 64.7%, European ancestry in the same region reaches 26.8%, while African ancestry reaches 0.85% (25). Peprah describes the lack of information on *FMR1* gene mutations in most countries with a non-European population (26). Further studies are needed to find out the extent of the mutation of the gene in the Pichincha Province.

The proportion of patients with FXS who came for genetic evaluation is low (2.7% of the total cases studied for rare diseases in HE1 since 2010) in relation to other diseases also diagnosed in the service such as Steinert myotonic dystrophy (12.2% of the cases studied) or Huntington's chorea (8.2% of the cases studied). This may be due to the lack of referral of patients with FXS and indicates a need for education of health professionals from different disciplines regarding the importance of early detection, which could lead to minimizing or preventing the risk of transmission.

In negative cases, there was a multimodal distribution of 27 (8.90%), 26 (7.28%), 29 (7.04%), and 35 (7.04%) repeats, similar to that reported in different populations worldwide where the allelic distribution of the expansion and the number of CGG repeats of the gene is between 29 and 30 (26) (Figure 3). The data are similar in studies conducted in Brazil (27), Cameroon (28), Chile (29), China and India (30), United States (31), and Mexico (32). The small differences found (one or two repeats of CGG) are related to experimental errors given by differences in the precision of the techniques used and the lack of their own population patterns (33), in addition to population and ethnic characteristics (34).

### Limitations

The standard procedure for the diagnosis of FXS is PCR and Southern blot, and results can only be analyzed with a gene analyzer or capillary electrophoresis equipment. Unfortunately, these tools were not available in the Medical Genetics service; therefore, only endpoint PCR and agarose gel electrophoresis were used. The absence of DNA fragments in the agarose gel indicated that the allele was expanded and was not observable due to the limited sensitivity of this technique (100–120 CGG repeats). In this case, the result was validated by real-time PCR to ensure adequate amplification of genetic material. In addition, the technique used cannot detect gene PM.

In addition, the sensitivity of the technique does not allow to visualize the second allele, corresponding to the homologous X chromosome in a woman; thus, there are two possibilities: two superimposed alleles with similar molecular weights. In this case, the patient would present a normal phenotype, without risk of transmission of FXS, or that the second allele had a large number of CGG repeats that cannot be amplified by the PCR. In this case, the patient would be a carrier of a PM or a complete mutation of the *FMR1* gene, with the risk of transmitting FXS to her descendants. To avoid confusion, this screening was not performed in women.

## Conclusion

Using the endpoint PCR technique, it is not possible to differentiate the cases of mutation carriers from those of PM, so it does not allow us to obtain a real rate of patients with the condition. It only allows us to make an approximation of the possible cases, functioning as an initial screening technique. Although our values were similar to those reported in the literature, as we were unable to differentiate the number of expansions, we could be overestimating the number of cases with a diagnosis of FXS. National strategies should be implemented to optimize molecular tests and establish proper protocols for the diagnosis to implement the use of diagnostic techniques such as SB and triplet-primed PCR assay, guides for management, and follow-up of patients, families, and communities at risk of presenting FXS.

The limitation of the study is the laboratory technique. The endpoint PCR and agarose gel electrophoresis used in this study do not allow to differentiate the premutated alleles (55 to 200 CGG repeats) and complete mutation alleles (>200 CGG repeats). These are important characteristics to understand the inheritance mechanism at the family (35). In addition to PCR, Southern blot and capillary electrophoresis are standard techniques for the diagnosis of FXS that allow confirmation of premutated allele carriers and full mutation. The laboratory does not have a capillary electrophoresis equipment because it is a complex and expensive technique. Instead, the Fast Start High Fidelity PCR System kit (Roche GmbH, Mannheim, Germany) was used to assemble the PCR and agarose gel electrophoresis. This study helps in the screening of the frequency of patients with FXS in Ecuador and makes visible the need to implement molecular diagnosis for people with intellectual disabilities and learning disorders.

## Data Availability Statement

The original contributions presented in the study are included in the article/Supplementary Material, further inquiries can be directed to the corresponding author/s.

## Ethics Statement

Ethical review and approval was not required for the study on human participants in accordance with the Local Legislation and Institutional Requirements. Written informed consent to participate in this study was provided by the participants' legal guardian/next of kin.

## Author Contributions

JP-P, EO, AL-C, and JV drafted the manuscript. AL-P, CR, and EO permormed testing and data collection.

## Conflict of Interest

The authors declare that the research was conducted in the absence of any commercial or financial relationships that could be construed as a potential conflict of interest.

## Publisher's Note

All claims expressed in this article are solely those of the authors and do not necessarily represent those of their affiliated organizations, or those of the publisher, the editors and the reviewers. Any product that may be evaluated in this article, or claim that may be made by its manufacturer, is not guaranteed or endorsed by the publisher.
