# Supplementary material for: Experiences of the Molecular Diagnosis of Fragile X Syndrome in Ecuador
Source: Front Psychiatry. 2021 Dec 13;12:716311. doi: 10.3389/fpsyt.2021.716311 (PMC8710471; doi:10.3389/fpsyt.2021.716311)
Supplement: Supplementary file 1 [file Data_Sheet_1.PDF]

	B	C	D	E	F	G	H	I	J	K	L
2	YEAR	N°	SEX	DIAGNOSIS AGE	LAB CODE	CH NUMBER	CLINICAL DIAGNOSIS	ORIGIN CITY	RESULTS	ALLELE WEIGHT	CGG REPETITION
3		1	M	11	ADN 092	442724	Fragile X	Quito	ND	300	27
4		2	M	5	ADN 127	386256	Fragile X	Pasaje	ND	300	27
5	2011	3	M	9	ADN 128	344450	Fragile X	Guayaquil	ND	300	27
6		4	M	6	ADN 162		Fragile X	Quito	ND	300	27
7		5	M	9	ADN 186		Fragile X	Quito	ND	300	27
8		6	M	9	ADN 194	453974	Fragile X	Quito, Carapungo	ND	350	43
9		7	M	8	ADN 217	353692	Fragile X	Quito	ND	350	43
10		8	M	11	ADN 223		Fragile X	Loja	ND	300	27
11		9	M	9	ADN 228	457250	Fragile X	Quito	ND	350	43
12	2012	10	M	13	ADN 238	458355	Fragile X	Quito	D		
13		11	M	6	ADN 250	460331	Fragile X	Quito	ND	300	27
14		12	M	7	ADN 268	377128	Develomental and Language Delay	Esmeraldas	ND	300	27
15		13	M	3	ADN 278	463979	Fragile X and Language Delay	Guayaquil	ND	320	33
16		14	M	2	ADN 275	430220	Fragile X	Quitumbe	ND	325	35
17		15	M	10	ADN 340	470456	ADHD	Guayas	ND	294	24
18		16	M	2	ADN 369	473369	Fragile X, developmental delay	Chillogallo	ND	325	35
19	2013	17	M	12	ADN 372	473890	Fragile X	Lago Agrio	ND	325	35
20		18	M	14	ADN 383	475481	Intelectual disability	Quito	ND	325	35
21		19	M	3	ADN 391	475930	Fragile X	Esmeraldas	ND	285	21
22		20	M	21	ADN 433	191019	Intelectual disability	Quito	ND	285	21
23		21	M	1	ADN 444	481600	Fragile X	Manta	ND	272	18
24		22	M	7	ADN 445	481717	Fragile X	Quito	ND	309	30
25		23	M	7	ADN 468	482976	Develomental and Language Delay	Manta	ND	309	30
26		24	M	5	ADN 490	484145	Intelectual disability	Guayaquil	ND	262	14
27	2014	25	M	2	ADN 523	485987	Intelectual disability	Quito	D		
28		26	M	8	ADN 528	486388	Intelectual disability	Ciudadela del Ejército	ND	269	16
29		27	M	14	ADN 540	487097	Intelectual disability	Sevilla Sucumbíos	ND	257	12
30		28	M	13	ADN 552	336881	Fragile X	Quito	ND	265	15
31		29	M	11	ADN 553	487825	Intelectual disability Fragile X	Quito	ND	269	16
32		30	M	6	ADN 618	491224	Intelectual disability	Quevedo	ND	314	31
33		31	M	15	ADN 681	494504	Motor delay	Guayaquil	ND	315	31
34	2015	32	M	3	ADN 702	495633	Intelectual disability	Quito - Carcelen	ND	325	34
35		33	M	13	ADN 704	436764	Other Congenital Malformations not classified	Ambato	ND	320	33
36		34	M	16	ADN 732	294626	Other Congenital Malformations not classified	Quito	ND	290	23
37		35	M	19	ADN 745	314697	Other Congenital Malformations not classified	Quito	ND	313	31
38		36	M	4	ADN 791	501056	Intelectual disability	Quito	ND	325	35
39		37	M	7	ADN 807	424350	ASD	Quito	D		
40		38	M	14	ADN 810	501706	Other Congenital Malformations not classified	Quito	ND	334	38
41		39	M	10	ADN 832	502500	Other Congenital Malformations not classified	Quito	ND	296	25
42		40	M	14	ADN 835	502672	Other Congenital Malformations not classified	Quito	ND	296	25
43		41	M	13	ADN 842	502751	Other Congenital Malformations not classified	Quito	ND	318	33
44		42	M	13	ADN 843	502753	Other Congenital Malformations not classified	Quito	ND	321	34
45		43	M	8	ADN 849	502898	Other Congenital Malformations not classified	Quito	ND	321	34
46		44	M	13	ADN 857	503179	Other Congenital Malformations not classified	Quito	ND	297	26
47		45	M	11	ADN 863	503261	Other Congenital Malformations not classified	Quito	ND	297	26
48		46	M	12	ADN 865	503237	Other Congenital Malformations not classified	Quito	ND	307	29
49		47	M	2	ADN 869	480959	Other Congenital Malformations not classified	Quito	ND	306	29
50	2016	48	M	9	ADN 898	503863	Other Congenital Malformations not classified	Quito	ND	302	27
51		49	M	24	ADN 899	462520	Epilepsy	Quito	ND	297	26
52		50	M	14	ADN 907	504056	Other Congenital Malformations not classified	Quito	ND	300	27
53		51	M	5	ADN 911	504157	Other Congenital Malformations not classified	Quito	ND	300	27
54		52	M	6	ADN 920	504579	Intelectual disability	Quito	ND	277	19
55		53	M	10	ADN 928	504841	Other Congenital Malformations not classified	Quito	ND	301	27
56		54	M	12	ADN 930	504577	Other Congenital Malformations not classified	Quito	ND	284	21
57		55	M	9	ADN 945	504812	Other Congenital Malformations not classified	Quito	ND	289	23
58		56	M	9	ADN 946	505294	Fragile X	Quito	D		
59		57	M	13	ADN 951	505759	Other Congenital Malformations not classified	Quito	ND	298	26
60		58	M	9	ADN 962	444305	ADHD	Quito	ND	299	26
61		59	M	5	ADN 978	452772	Fragile X	Sangolqui	D		
62		60	M	16	ADN 987	507117	Other Congenital Malformations not classified	Carchi	ND	318	32
63		61	M	12	ADN 1046	509264	Other Congenital Malformations not classified	Quito	ND	333	37
64		62	M	14	ADN 1047	509280	Other Congenital Malformations not classified	Quito	ND	271	16
65		63	M	10	ADN 1048	509382	Other Congenital Malformations not classified	Pedro Vicente Maldonado	ND	296	25
66		64	M	20	ADN 1051	229350	Other Congenital Malformations not classified	Quito	ND	288	22
67		65	M	4	ADN 1064	468821	Develomental and Language Delay		ND	285	22
68		66	M	13	ADN 1077	510244	Other Congenital Malformations not classified		ND	303	28
69		67	M	5	ADN 1078	510200	ADHD		ND	303	28
70		68	M	13	ADN 1100	509822	Fragile X		ND	304	28
71		69	M	15	ADN 1103	428031	Other Congenital Malformations not classified		ND	304	28
72		70	M	4	ADN 1112	489784	ASD		ND	314	31
73		71	M	15	ADN 1117	313743	ADHD		ND	325	35
74		72	M	12	ADN 1132	510397	Other Congenital Malformations not classified		ND	293	24
75		73	M	6	ADN 1139	508546	Other Congenital Malformations not classified		ND	290	23
76		74	M	2	ADN 1154	511372	ASD		ND	307	29
77		75	M	7	ADN 1102	511057	Fragile X		ND	316	32
78		76	M	17	ADN 1177	512550	Other Congenital Malformations not classified		ND	306	29
79		77	M	9	ADN 1179	433568	ASD		ND	304	29
80		78	M	6	ADN 1184	511446	Other Congenital Malformations not classified		ND	294	25
81		79	M	2	ADN 1198	512629	ASD		ND	273	18
82		80	M	17	ADN 1199	512904	Other Congenital Malformations not classified		ND	305	28
83		81	M	6	ADN 1225	513410	Language delay		ND	305	28
84		82	M	13	ADN 1226	513461	Fragile X		ND	291	24
85	2017	83	M	2	ADN 1231	495874	Macrocephaly<br>lia		ND	291	24
86		84	M	3	ADN 1234	510361	Other Congenital Malformations not classified		ND	311	30
87		85	M	7	ADN 1238	513676	Fragile X		ND	291	23
88		86	M	8	ADN 1243	513739	Fragile X		ND	296	25
89		87	M	15	ADN 1251	323328	Intelectual Disability		ND	314	31
90		88	M	7	ADN 1253	513962	Fragile X		ND	314	31
91		89	M	2	ADN 1255	492911	Language delay		ND	310	30
92		90	M	10	ADN 1258	513959	Fragile X		ND	309	30
93		91	M	5	ADN 1270	514190	Other Congenital Malformations not classified		ND	278	19
94		92	M		ADN 1274	514239	Fragile X		D		
95		93	M	9	ADN 1278	514360	Other Congenital Malformations not classified		ND	314	31
96		94	M	9	ADN 1287	513457	Intelectual Disability		ND	290	23
97		95	M	24	ADN 1303	514815	Fragile X		ND	295	25
98		96	M	7	ADN 1305	514819	Fragile X		D		
99		97	M	9	ADN 1306	514818	Fragile X		D		
100		98	M	13	ADN 1311	514854	Fragile X		D		
101		99	M	16	ADN 1312	514855	Fragile X		D		
102		100	M	3	ADN 1318	510067	ASD		ND	325	35
103		101	M	9	ADN 1319	514937	Other Congenital Malformations not classified		ND	325	35
104		102	M	1	ADN 1322	513875	Other Congenital Malformations not classified		ND	322	34
105		103	M	0	ADN 1364	515581	Fragile X		ND	322	34
106		104	M	10	ADN 1372	468312	ASD		ND	294	25
107		105	M	8	ADN 1375	515640	Fragile X		ND	294	25
108		106	M	1	ADN 1386	515927	Developmental delay		ND	309	29
109		107	M	16	ADN 1398	513693	ASD		ND	305	29
110		108	M	22	ADN 1411	516188	ASD		ND	343	41
111		109	M	20	ADN 1412	516189	ASD		ND	310	30
112		110	M	17	ADN 1415	516264	Fragile X		D		
113		111	M	17	ADN 1416	516263	Fragile X		D		
114		112	M	16	ADN 1418	516298	Intelectual Disability		ND	286	22
115		113	M	1	ADN 1422	516338	Epilepsy		ND	292	24
116		114	M	1	ADN 1428	516412	Other Congenital Malformations not classified		ND	298	26
117		115	M	2	ADN 1429	495718	ASD		ND	298	26
118		116	M	3	ADN 1467	517008	ASD		ND	289	23
119		117	M	1	ADN 1476	503877	Motor delay		ND	310	30
120		118	M	7	ADN 1484	517371	Fragile X		D		
121		119	M	2	ADN 1486	517552	Fragile X		ND	307	29
122		120	M	11	ADN 1506	396307	Intelectual Disability		ND	277	19
123		121	M	9	ADN 1528	420432	Epilepsy		ND	301	27
124		122	M	8	ADN 1529	518110	Fragile X		ND	313	31
125		123	M	11	ADN 1535	SOCIAL	Intelectual disability		ND	307	29
126		124	M	8	ADN 1540	431209	ASD		ND	307	29
127		125	M	9	ADN 1571	518803	Other Congenital Malformations not classified		ND	307	29
128		126	M		ADN 1574	517891	Epilepsy		ND	307	29
129		127	M	16	ADN 1601	519211	ASD		ND	298	26

	B	C	D	E	F	G	H	I	J	K	L
130		128	M	13	ADN 1602	519235	ASD		ND	301	27
131		129	M	7	ADN 1615	473884	Intelectual Disability		ND	319	33
132	2018	130	M	2	ADN 1616	498441	Behavior Concerns		ND	304	28
133		131	M	11	ADN 1625	3137468	ASD		ND	304	28
134		132	M	14	ADN 1627	519527	Balanced Rearrangements and Structural Markers, not elsewhere classified		ND	316	32
135		133	M	13	ADN 1637	519632	Other Congenital Malformations not classified		ND	292	24
136		134	M	5	ADN 1638	488500	Developmental delay		ND	322	34
137		135	M	5	ADN 1655	519907	Fragile X		ND	292	24
138		136	M	16	ADN 1660	519989	Fragile X		ND	289	23
139		137	M	3	ADN 1690	520518	Fragile X		ND	298	26
140		138	M	7	ADN 1692	520585	Fragile X		ND	298	26
141		139	M	15	ADN 1694	520597	Other Congenital Malformations not classified		ND	295	25
142		140	M	0	ADN 1707	520744	Defects in post-translational modification of lysosomal enzymes		ND	298	26
143		141	M	11	ADN 1708	520743	Other Congenital Malformations not classified		ND	298	26
144		142	M	3	ADN 1712	520836	Fragile X		ND	304	28
145		143	M	2	ADN 1731	506843	ASD		ND	274	18
146		144	M	13	ADN 1737	355091	Learning Dificulties		ND	289	23
147		145	M	6	ADN 1739	521269	Fragile X		ND	291	24
148		146	M	12	ADN 1745	521357	Fragile X		ND	295	25
149		147	M	18	ADN 1755	521543	Intelectual disability		ND	289	23
150		148	M	9	ADN 1761	521743	Fragile X		ND	262	14
151		149	M	12	ADN 1762	488203	Intelectual disability		ND	313	31
152		150	M	2	ADN 1764	515488	ASD		ND	301	27
153		151	M	15	ADN 1772	346596	Epilepsy		ND	316	32
154		152	M	14	ADN 1779	388704	ASD		ND	295	25
155		153	M	3	ADN 1804	501276	Behavior Concerns		ND	319	33
156		154	M	3	ADN 1805	512408	ASD		ND	286	22
157		155	M	2	ADN 1811	510733	ASD		ND	286	22
158		156	M	2	ADN 1813	522915	Developmental delay		ND	262	14
159		157	M	3	ADN 1814	494090	Developmental delay		ND	259	13
160		158	M	3	ADN 1819	493599	ASD		ND	305	28
161		159	M	4	ADN 1827	523235	Other Congenital Malformations not classified		ND	293	25
162		160	M	12	ADN 1840	523392	Other Congenital Malformations not classified		ND	304	28
163		161	M	18	ADN 1843	523331	Other Congenital Malformations not classified		ND	295	25
164		162	M	3	ADN 1848	499611	Developmental delay		ND	295	25
165		163	M	6	ADN 1858	523595	ASD		ND	307	29
166		164	M	4	ADN 1862	523701	Fragile X		ND	313	31
167		165	M	2	ADN 1882	517229	Epilepsy		ND	325	35
168		166	M	5	ADN 1889	523909	Fragile X		ND	302	27
169		167	M	6	ADN 1901	460853	Language delay		ND	319	33
170		168	M	2	ADN 1911	523941	ASD		ND	328	36
171		169	M	11	ADN 1914	524243	Intelectual Disability		ND	325	35
172		170	M	15	ADN 1916	524244	Fragile X		ND	340	40
173		171	M	11	ADN 1924	524402	Fragile X		ND	307	29
174		172	M	8	ADN 1927	524434	Intelectual Disability		ND	298	26
175		173	M	4	ADN 1928	524406	Fragile X		ND	298	26
176		174	M	5	ADN 1934	524497	ASD		ND	307	29
177		175	M	6	ADN 1935	476302	ASD		ND	313	31
178		176	M	5	ADN 1939	524590	Intelectual Disability		ND	325	35
179		177	M	4	ADN 1946	524745	ASD		ND	337	39
180		178	M	5	ADN 1951	501283	ASD		ND	334	38
181		179	M	11	ADN 1961	524951	Intelectual Disability		ND	331	37
182		180	M	6	ADN 1963	525017	Intelectual Disability		D		
183		181	M	7	ADN 1967	525077	Intelectual Disability		ND	331	37
184	2019	182	M	17	ADN 1968	525078	Intelectual Disability		ND	322	34
185		183	M	13	ADN 1970	SOCIAL			ND	316	32
186		184	M	12	ADN 1982	525269	Fragile X		ND	322	34
187		185	M	5	ADN 1991	525376	Fragile X		D		
188		186	M	3	ADN 2002	525184	ASD		ND	325	35
189		187	M	7	ADN 2017	524639	Intelectual Disability		ND	298	26
190		188	M	3	ADN 2025	525737	Fragile X		ND	301	27
191		189	M	2	ADN 2027	524266	Language delay		ND	313	31
192		190	M	3	ADN 2050	502125	Epilepsy		ND	277	19
193		191	M	7	ADN 2052	524355	Epilepsy		ND	319	33
194		192	M	6	ADN 2055	526204	ASD		ND	331	37
195		193	M	14	ADN 2059	526237	Intelectual Disability		ND	346	42
196		194	M	3	ADN 2073	523753	ASD		ND	328	36
197		195	M	6	ADN 2074	526052	Developmental delay		ND	340	40
198		196	M	3	ADN 2085	526387	ASD		ND	319	33
199		197	M	2	ADN 2089	526793	Developmental delay		ND	325	35
200		198	M	15	ADN 2096	379631	Intelectual Disability		ND	322	34
201		199	M	21	ADN 2102	525999	Fragile X		ND	313	31
202		200	M	59	ADN 2020	525639	Myotonic disorders		ND	346	42
203		201	M	12	ADN 2104	399790	ADHD		ND	319	33
204		202	M	6	ADN 2107	526544	Behavior Concerns		ND	292	24
205		203	M	9	ADN 2108	526953	Fragile X		ND	334	38
206		204	M	7	ADN 2112	527079	Fragile X		ND	301	27
207		205	M	3	ADN 2123	510132	Language delay		ND	331	37
208		206	M	3	ADN 2124	526540	Stereotyped movements		ND	334	38
209		207	M	11	ADN 2132	527442	Huntington disease		ND	322	34
210		208	M	5	ADN 2135	527512	Intelectual Disability		ND	310	30
211		209	M	1	ADN 2136	517168	Intelectual Disability		ND	313	31
212		210	M	3	ADN 2139	527569	ASD		ND	316	32
213		211	M	2	ADN 2141	525195	Arteriovenous malformation of the cerebral vessels		ND	310	30
214		212	M	11	ADN 2143	527659	Other Congenital Malformations not classified		D		
215		213	M	13	ADN 2154	527915	Developmental delay		ND	268	16
216		214	M	9	ADN 2162	528006	Intelectual Disability		ND	298	26
217		215	M	6	ADN 2174	528266	Fragile X		ND	320	34
218		216	M	14	ADN 2176	528271	Fragile X		ND	330	36
219		217	M	16	ADN 2177	528269	Fragile X		ND	347	42
220		218	M	4	ADN 2185	500261	ASD		ND	335	38
221		219	M		ADN 2189		Intelectual disability		D		
222		220	M		ADN 2194		Intelectual Disability		D		
223		221	M	11	ADN 2198	528452	Fragile X		D		
224		222	M	4	ADN 2203	528693	Fragile X		ND	289	23
225		223	M	8	ADN 2211	528487	Fragile X		ND	301	27
226	2020	224	M	3	ADN 2212	528883	ASD		ND	304	28
227		225	M	12	ADN 2215	512020	ASD		ND	310	30
228		226	M	12	ADN 2216	528901	Fragile X		ND	325	35
229		227	M	9	ADN 2222	460258	Café au lait spots		ND	292	24
230		228	M	2	ADN 2226	529074	Fragile X		ND	310	30
231		229	M	3	ADN 2227	527760	Language delay		ND	307	29
232		230	M	8	ADN 2238	529539	Other Congenital Malformations not classified		ND	301	27
233		231	M	13	ADN 2259	468312	Intelectual Disability		ND	322	34
234		232	M		ADN 2267	SOCIAL	Suspicion of Adrenoleukodystrophy		ND	304	28
235		233	M	11	ADN 2301	530096	Developmental delay		ND	328	36
236		234	M	39	ADN 2321	530353	Motor neuron diseases		ND	289	23
237		235	M	3	ADN 2349	530683	Motor delay		ND	325	35
238		236	M	2	ADN 2358	530652	Child brain paralysis		ND	292	24
239		237	M	19	ADN 2368	310775	Learning Dificulties		ND	322	34
240		238	M	2	ADN 2373	526370	ASD		ND	322	34
241		239	M	58	ADN 2380	61529	Parkinson Disease		ND	274	18
242		240	M	5	ADN 2389	500606	Language delay		D		
243	2021	241	M	14	ADN 2400	418354	ADHD		ND	311	30
244		242	M	3	ADN 2404	516305	Language delay		ND	325	35
245		243	M	3	ADN 2417	518406	Epilepsy		ND	295	25
246		244	M	7	ADN 2397	476293	Language delay		ND	298	26
247		245	M	2	ADN 2420	531294	Language delay		ND	302	27
248		246	M	2	ADN 2421	531295	Language delay		ND	302	27
249		247	M	4	ADN 2422	531376	ASD		ND	300	26
250										**306**	**29**
